# Ulnar Polydactyly: Family History, Phenotypes, Associated Anomalies, and Surgical Treatment in a Prospective 7-Year Registry Study

**DOI:** 10.1016/j.jhsg.2026.101170

**Published:** 2026-11-03

**Authors:** Elias Tallay, Ishanee Nirmalanathan, Mona Irene Winge, Jarkko Jokihaara, Ida Neergård Sletten

**Affiliations:** ∗ Division of Surgery, Østfold Hospital Trust, Grålum, Norway; † Division of Orthopaedic Surgery, Oslo University Hospital, Oslo, Norway; ‡ Department of Hand Surgery, Tampere University Hospital, Central Hospital, Tampere, Finland; § Faculty of Medicine and Health Technology, Tampere University, Tampere, Finland

**Keywords:** Associated anomalies, Congenital upper limb anomaly, Phenotype classification, Registry study, Ulnar polydactyly

## Abstract

**Purpose:**

Ulnar polydactyly (UP) is a common congenital upper limb anomaly, yet prospective data on inheritance patterns, phenotypic distribution, associated anomalies, and treatment outcomes remain limited.

**Methods:**

We conducted a registry-based observational study of consecutive children with UP enrolled in a prospectively maintained congenital upper limb anomaly registry at a tertiary referral center between 2018 and 2025. Data included family history, pregnancy characteristics, Congenital Upper Limb Differences database classification of phenotypes (A1–A6 and B), laterality, associated anomalies and genetically verified syndromes, and interventions. We analyzed the strength of associations between hand presentation and associated anomalies using multivariable logistic regression.

**Results:**

We included 98 patients with 160 affected hands. Fifty-four percent had a non-Nordic family background, substantially exceeding the proportion of individuals with a non-Nordic background in the general population. Forty-two percent reported a positive family history of UP. Type B UP predominated (85%). Associated lower limb anomalies occurred in 24% of patients. Phenotypes A and bilateral affection showed strong associations with lower limb anomalies and genetic syndromes and/or anomalies besides the limbs. Our unit performed all surgeries without any complications, yet we reoperated many patients after inadequate ligation of type B UP in the maternity wards.

**Conclusions:**

Ulnar polydactyly comprises phenotypic subgroups with differing risks of associated anomalies, supporting a phenotype-based approach to clinical assessment. We recommend genetic evaluation for all patients with phenotype A and for those with bilateral type B with associated lower limb anomalies. In contrast, unilateral and bilateral type B without lower limb involvement or associated anomalies on routine clinical examination do not require further evaluation.

**Type of study/level of evidence:**

Clinical II.

Ulnar polydactyly (UP) is a common congenital upper limb anomaly (CULA), with considerable regional and ethnic differences in prevalence; in some populations, it is the most frequently observed CULA.^1^ The malformation occurs early in fetal development and presents unilaterally or bilaterally as an isolated anomaly or associated with other congenital anomalies or syndromes. The development of the radial–ulnar axis in the limb bud is regulated by the zone of polarizing activity, and polydactyly has been associated with abnormal sonic hedgehog (Shh) signaling due to mutations in the *Shh* or *GLI1* genes.^2^ Ulnar polydactyly is classified as IB2vi in the Oberg–Manske–Tonkin classification,^3^ and by the Temtamy–McKusick classification from 1978 into type A, representing a more or less fully developed sixth digit, and type B, characterized by a rudimentary floating digit connected to the fifth ray by a skin pedicle.^4^ Among several proposed subclassification systems for type A, the Pritsch–Oishi system from 2013 is the most widely recognized.^5^ In 2025, the American Congenital Upper Limb Differences database (CoULD) Study Group demonstrated almost perfect inter- and intraobserver reliability for a new classification designed to capture all subtypes and incorporate an intuitive progression of subtypes.^6^

The treatment of UP is guided by its phenotype. Type A typically requires reconstructive surgery at 1–2 years of age, with the transfer of functional structures, such as extensor tendons and ligaments, from the minor digit to the remaining more developed ulnar finger. Type B is more commonly managed with a simple excision by hand surgeons or ligation by pediatricians or nursing staff in the neonatal period. A recent meta-analysis reported generally favorable outcomes across treatment modalities for UP type B, but the included studies were of low methodological quality, underscoring the need for prospective observational research.^7^

Despite the high prevalence of UP, limited data are available on family history and the frequency and spectrum of associated anomalies, particularly for type B. Limb anomalies are often the most obvious, and hand surgeons should be aware of associated internal organ anomalies and syndromes to ensure these have been adequately ruled out. The aim of this study was to investigate parental, gestational, and infant characteristics, including associated anomalies and syndromes, in children with UP, as well as the surgical procedures performed.

## Materials and Methods

This cohort study used prospectively collected data from the CULA North Oslo Registry on consecutive children with UP enrolled between May 8, 2018, and May 7, 2025. This research registry includes patients with congenital upper limb anomalies and records family and pregnancy information, as well as diagnostic and treatment data. Parents/guardians of all included patients provided written broad consent for the use of their data in research studies at the time of registry enrollment. The Regional Committees for Medical and Health Research Ethics–South East Norway (ID 910396) and the Data Protection Officer at Oslo University Hospital (ID 25/15084) approved the study. It was conducted in accordance with the Declaration of Helsinki and the Strengthening the Reporting of Observational Studies in Epidemiology statement.^8^, ^9^

The parents reported national family background. Non-Nordic ethnic background was defined as the patient, a parent, or a grandparent originating from a country outside Denmark, Finland, Iceland, Norway, and Sweden. This definition is broader than the official Statistics Norway category of immigrants and Norwegian-born individuals with immigrant parents, which comprised 21% of the Norwegian population in 2026.^10^ Other parent-reported variables included in the study were family history of congenital anomalies, maternal and paternal age at the patient’s birth, folic acid supplement use during pregnancy, assisted reproductive technology (ART), infant gestational age, and birth weight. The assessor-reported study outcomes were sex, age at referral and at surgery, CoULD classification of UP (Table 1), unilateral or bilateral affection, associated anomalies, genetically verified syndromes, interventions, and eventual complications. We prospectively classified patients using the Pritsch–Oishi system and then retrospectively reclassified and reported them according to the new CoULD system.

**Table 1:** The CoULD UP Classification

Phenotype	Description
Type A	A well-developed accessory digit with a substantial soft-tissue bridge or bony articulation.
A1-hypoplastic	Markedly hypoplastic, typically triphalangeal, does not articulate with the metacarpal but has a soft-tissue bridge exceeding 50% of the digital diameter.
A2-phalangeal	The phalanges are partially duplicated, and the metacarpal is normal.
A3-divergent metacarpophalangeal	Two triphalangeal postaxial digits articulate with each other, and the metacarpal and the two fingers diverge at the metacarpophalangeal joint.
A4-bifid metacarpal	The fifth metacarpal has two distinct articular surfaces, and the two postaxial fingers are straight.
A5-duplicated metacarpal	The metacarpal is also duplicated, well-developed or rudimentary.
A6-complex	Combination of subtypes.
Type B	Skin nub or rudimentary pedunculated digit with a narrow soft tissue stalk less than 50% of the digital diameter.

CoULD, Congenital Upper Limb Differences database.

### Statistics

We presented categorical variables as frequencies and percentages**,** and continuous variables as means with confidence intervals or medians with interquartile ranges (IQRs), depending on data distribution. Because of small observed cell counts in the phenotype contingency tables, we combined the A phenotypes and examined group differences using Fisher exact test rather than the Pearson chi-square test. We assessed the magnitude of associations between phenotypes (A vs B) and laterality with other anomalies and syndromes using logistic regression. Two outcomes were analyzed: (1) presence of lower limb anomalies and (2) presence of a syndrome and/or anomalies besides the limbs. For each outcome, we first performed univariable logistic regression, followed by multivariable models including both predictors (phenotype and laterality). Phenotype B and unilateral affection were chosen as references in all models. Results were presented as odds ratios with 95% confidence intervals (CIs). A two-sided *P* value of <.05 was considered statistically significant.

## Results

During the study period, we collected background information and written consent for 98 of 112 (88%) consecutive patients with UP. Fifty-two (53%) of the 98 included patients were female. The median age at first presentation was 45 days (IQR: 27–105). Fifty-three patients (54%) had a non-Nordic background, with individuals of Sub-Saharan African origin comprising the largest subgroup. The parents reported a positive family history of UP in 41 (42%) patients. The mean maternal and paternal ages at delivery were 31 (95% CI: 30–32) and 34 years (95% CI: 33–35), respectively. Eighty-one of 94 mothers (86%) reported folic acid supplementation during pregnancy. Seven children (7%) were conceived following ART; all had bilateral UP (2 type A and 5 type B).

The median gestational length was 40 weeks (IQR: 38–41), and the mean birth weight was 3,561 g (95% CI: 3,466–3,656). Bilateral UP type B represented the most common phenotype (Table 2). Among type A cases, CoULD subtypes A1 and A4 were most frequent (Table 2). Of 36 patients with unilateral presentation, 29 had left-sided affection.

**Table 2:** Ulnar Polydactyly Phenotypes (n = 98 Patients)

	Phenotype	Patients (n)	Hands (n)
Unilateral	A1	1	1
	A4	3	3
	B	32	32
Bilateral	A1 + A1	4	8
	A1 + A4	1	2
	A4 + A4	1	2
	A4 + A5	1	2
	A5 + A5	1	2
	A1 + B	1	2
	A3 + B	1	2
	A4 + B	2	4
	B + B	50	100
Type A, total		16	24
Type B, total		86	136
**Total**		**98**	**160**

Six of the 98 patients had associated CULA, all with bilateral UP (1 type A and 5 type B). Three of them had GLI3-associated polydactyly or Greig cephalopolysyndactyly with bilateral Wassel type 1 radial polydactyly. Two other syndromic cases had bilateral finger brachydactyly, and one of them also had bilateral simple syndactyly of the third web (Table 3). One had bilateral osseous syndactyly of the fourth web, and one had unilateral simple syndactyly of the second web.

**Table 3:** Genetically Verified Syndromes (n = 8 Patients)

Syndrome	Affected Gene	Associated Anomalies and Conditions∗
Bardet–Biedl	BBS10	Abnormal obesity, postaxial polydactyly of the feet
CEP19-related condition	CEP19	Abnormal obesity, postaxial polydactyly feet
GLI3-associated polydactyly	GLI3	Radial/preaxial polydactyly hands, preaxial polydactyly feet, syndactyly toes
Greig cephalopolysyndactyly	GLI3	Characteristic facial features, agenesis of the corpus callosum, radial/preaxial polydactyly hands, postaxial polydactyly feet
KBG syndrome	ANKRD11	Characteristic facial features, developmental delay, postaxial polydactyly feet
Pfeiffer type 1	FGFR1	Characteristic facial features, brachycephaly, cranial synostosis, ASD, brachydactyly of the hands, syndactyly of the hands and toes
Short-rib thoracic dysplasia 15 with polydactyly	DYNC2LI1	Esophageal atresia, tracheoesophageal fistula, tracheomalacia, coarctation of the aorta, ASD, VSD, brachydactyly hands and feet, postaxial polydactyly feet

ASD, atrial septal defect; VSD, ventricular septal defect.

∗ Other than UP hand(s).

Congenital lower limb anomalies (CLLAs) were present in 24 patients, exclusively as toe anomalies, including polydactyly (20), syndactyly (2), combined polydactyly and syndactyly (1), and overriding toes (1). Six syndromes were identified in eight patients, and their UP phenotypes were unilateral A (1), bilateral A (3), or bilateral B (4). All eight patients had associated CLLA (Table 3). Patients with phenotype A and/or bilateral UP affection had more associated anomalies (Tables 4 and 5). Among six patients with phenotype A1, A1+A1, or A1+B, two had a syndrome or internal organ anomalies, while this was present in 4 of 10 patients who had phenotypes A2–A5. Eight patients had anomalies in organ systems other than the limbs, including craniomaxillofacial (3), neurological (2), cardiac (4), genitourinary (2), gastrointestinal (2), and airway/pulmonary (2) anomalies. All these patients also had CLLA, except for one patient with bilateral UP type B, hypospadias, and an umbilical hernia. Multivariable regression analyses revealed strong associations between associated anomalies and patients with phenotype A and/or bilateral affection, although the wide confidence intervals indicated some uncertainty (Table 6).

**Table 4:** Ulnar Polydactyly Phenotypes A and B and Associated Anomalies (n = 98 Patients)

	Phenotype					*P*∗
	A (n = 4)	A+A (n = 8)	A+B (n = 4)	B (n = 32)	B+B (n = 50)	
Hand anomalies only (n = 73)	2	0	2	31	38	<.001
Lower limb anomalies† (n = 24)	2	8	2	1	11	<.001
Other anomalies‡ (n = 8)	0	3	0	0	5	.120
Genetically confirmed syndromes (n = 8)	1	3	0	0	4	.023

∗ Fisher exact test for combined groups phenotypes A, A+A, and A+B versus combined groups phenotype B and B+B.

† Polydactyly, syndactyly, and overriding toes.

‡ Combinations of craniomaxillofacial, neurological, cardiac, genitourinary, gastrointestinal, and airway/pulmonary anomalies.

**Table 5:** Ulnar Polydactyly Laterality and Associated Anomalies (n = 98 Patients)

	Unilateral A or B (n = 36)	Bilateral A+A, A+B, or B+B (n = 62)	*P*∗
Hand anomalies only (n = 73)	33	40	.003
Lower limb anomalies (n = 24)†	3	21	.006
Other anomalies (n = 8)‡	0	8	.025
Genetically confirmed syndromes (n = 8)	1	7	.251

∗ Fisher exact test.

† Polydactyly, syndactyly, and overriding toes.

‡ Combinations of craniomaxillofacial, neurological, cardiac, genitourinary, gastrointestinal, and airway/pulmonary anomalies.

**Table 6:** Associations Between UP Phenotypes (A vs B and Laterality) and Associated Anomalies (n = 98 Patients)

Outcome	Events	Predictor	Crude OR (95% CI)	*P*	Adjusted OR (95% CI)	*P*
Lower limb anomalies	24	Type A (ref: B)	17.5 (4.8–63.4)	<.001	20.7 (4.85–88.2)	<.001
		Bilateral (ref: unilateral)	5.6 (1.55–20.5)	.009	7.02 (1.51–32.7)	.013
Anomalies besides limbs and/or syndromes	12	Type A (ref: B)	7.60 (2.05–28.2)	.002	7.06 (1.81–27.5)	.005
		Bilateral (ref: unilateral)	7.55 (0.93–61.1)	.058	6.91 (0.81–58.7)	.077

OR, odds ratio.

Seventeen patients underwent ligation of the ulnar digits at the maternity ward; 12 of these required reoperations in our unit because of inadequate primary treatment. All patients referred with untreated UP type B underwent surgical excision under local anesthesia supplemented by oral sucrose at a median age of 35 days (IQR: 22–61 days). Parents declined surgery for two patients with phenotype A. We observed no surgical complications in our unit.

## Discussion

In this observational study of 98 consecutive UP patients, 54% had a non-Nordic family background, and 42% had a positive family history of UP. Bilateral type B was the most common phenotype, followed by unilateral type B. Unilateral type B UPs were never associated with anomalies outside the musculoskeletal system. In bilateral UP, associated anomalies were common and affected most frequently the lower limbs. Syndromic UPs were rare. Phenotype A, including the new CoULD phenotype A1 previously often classified as type B, had a higher rate of associated anomalies and syndromes compared to type B. Surgical treatment in our unit was uncomplicated for all patients, but many had been referred because of inadequate ligation of type B UPs in maternity wards.

The main strength of this study is the use of a prospective registry, ensuring systematic data collection on associated anomalies. The relatively long inclusion period and high research registry enrollment rate strengthen the study’s internal validity. Secondarily, we have quantified the risk of associated anomalies and syndromes in UP type A and B using regression modeling. Important limitations include the single-center study design, which may limit generalizability, and the relatively small number of patients with UP type A and events for the combined outcome of syndrome and/or anomalies besides the limbs, which restricted the precision of the estimates. However, the direction and magnitude of the associations were consistent across outcomes, and the predictors were based on clinical relevance.

The proportion of patients with a non-Nordic ethnic background markedly exceeded the national proportion of individuals with an immigrant background. Almost half of the patients reported a positive family history of UP, supporting a strong hereditary contribution. International reports underscore the wide geographic variation in UP prevalence and phenotype. In a cohort from Saudi Arabia, the incidence of UP was approximately 1 in 1,000 live births and 11% of patients had a positive family history,^11^ the latter much lower than in our study. Recent studies from East Asia suggest that UP is considerably less common in the Chinese and Japanese populations and may exhibit distinct patterns of associated anomalies, particularly a high rate of associated lower limb polydactyly.^12^, ^13^ The above differences highlight the influence of ancestral genetic background on both the frequency and expression of UP. No conclusions could be drawn regarding folic acid supplementation or ART. Folic acid supplementation rates were similar to those reported in the general population.^14^ Although approximately 5% of children in Norway are conceived using ART,^15^ the small number of ART-conceived children in our cohort precluded any assessment of a potential association with UP.

A majority of the 160 hands (85%) were classified as type B, consistent with the finding of 75% in a large population-based study from Mexico.^16^ Among type A hands, CoULD subtypes A1 and A4 were the most frequent, in line with the findings from the CoULD Registry.^6^ Bilateral affection was most common for both type A and B, and the combination of type A and B was rare. Our finding of a predominance of left-sided involvement in unilateral UP has been reported previously,^16^, ^17^ but as the data are inconsistent,^18^ this warrants further investigation.

Patients with UP type A exhibited a distinct clinical profile, with 75% showing bilateral involvement and 75% having associated anomalies in the lower limbs or internal organs. This pattern suggests that more complex or bilateral upper limb phenotypes reflect broader disruptions in limb development. Through regression analyses, we found strong associations between associated anomalies and type A and bilateral affection. The associations were strongest for CLLA, where the adjusted odds ratios exceeded the univariable estimates, indicating positive confounding due to the correlation between type A and bilateral involvement. Syndromes and internal organ anomalies categories were small and overlapping, and were therefore analyzed as a single outcome. For this combined outcome, the adjusted estimates were slightly lower than the univariable values, consistent with negative confounding. This suggests that part of the univariable association was attributable to overlap between the two predictors. The presence of both positive and negative confounding across outcomes highlights the importance of multivariable modeling, even when only two clinically relevant predictors are included. To our knowledge, no previous studies have examined whether syndromic UP occurs exclusively in specific phenotypes, as we observed only in patients with unilateral type A or bilateral type A or B, all of whom also had a CLLA. Likewise, prior literature has not assessed the co-occurrence of internal organ anomalies and CLLA; in our cohort, all such patients had CLLA, except for one who had other externally detectable anomalies. Together, these findings suggest that in patients without phenotype A or associated CLLA, further evaluation for internal organ anomalies or syndromes is unlikely to be necessary.

The classification of UP continues to evolve. Early systems focused on genetic inheritance patterns or broad distinctions between rudimentary and fully developed digits, whereas later classifications increasingly emphasized skeletal anatomy and surgical complexity.^6^, ^13^, ^19^ Beyond facilitating communication and surgical planning, our findings suggest that phenotypic classification may also provide prognostic information regarding the risk of associated anomalies. We found the new CoULD system intuitive and clinically useful, and reclassification of all cases was feasible. The system captures the A1 subtype, which we had previously classified as type B in most cases for lack of a specified alternative in the Pritsch–Oishi system. Based on our finding of a similar proportion of associated anomalies and syndromes across all phenotype A subtypes, this distinction is important as it reflects a different clinical profile.

Based on our findings, we recommend genetic evaluation for all patients with phenotype A regardless of laterality and for patients with bilateral type B and associated CLLA. Although infants are typically assessed by pediatricians in the maternity ward before referral to hand units, congenital hand surgeons should ensure that such patients have undergone appropriate evaluations to detect internal organ anomalies and syndromes early. Future studies should prioritize multicenter, population-based designs to better define prevalence and associated anomalies of all UP subtypes across demographic groups in large prospective cohorts and to identify genetic variants underlying associated anomalies and syndromes.

## Declaration of Generative AI and AI-Assisted Technologies in the Writing Process

During the preparation of this work, the authors used AI-assisted language tools (Grammarly and Microsoft Copilot) in order to support grammar correction and improve readability during the editing phase of the manuscript. After using these tools, the authors reviewed and edited the content as needed and take full responsibility for the content of the publication.

## Conflicts of Interest

No benefits in any form have been received or will be received related directly to this article.
